# Evaluation of Intermittent Restricted Eating Using the Interval Weight Loss Online Platform in an Everyday Setting

**DOI:** 10.3390/nu17020332

**Published:** 2025-01-17

**Authors:** Marijka Batterham, Bradley Wakefield

**Affiliations:** 1 Statistical Consulting Centre, National Institute for Applied Statistics Research Australia, School of Mathematics and Applied Statistics, University of Wollongong, Wollongong, NSW 2522, Australia; bradleyw@uow.edu.au; 2 Health Innovations, Faculty of Science, Medicine and Health, University of Wollongong, Wollongong, NSW 2522, Australia

**Keywords:** online weight loss, intermittent restricted eating, commercial weight loss program

## Abstract

**Background/Objectives**: Obesity remains a global health challenge. Many commercial online weight loss programs are available, and they have advantages in terms of scalability and access. Few of these programs have been evaluated for effectiveness in a real-world context. This study reports on the weight loss achieved, platform engagement, and characteristics of successful weight loss predictions in subscribers to the Interval Weight Loss (IWL) program. The Interval Weight Loss program promotes intermittent restricted eating in addition to lifestyle changes in diet composition, exercise, and sleep. **Methods**: Data for 1705 adults subscribing to the program for >30 days between 2019 and 2024 were included in the analysis. A linear mixed model with polynomial terms was used to model weight loss over time with interaction terms for gender and age. Survival analysis was used to model the proportions and time frame of those meeting 2%, 5%, and 10% weight loss targets and the proportion meeting their goal weight. The focus of the analysis was on the effect at 365 days. Descriptive data from a subset of participants (n = 205) who completed a questionnaire about change in lifestyle habits and mood are also presented. **Results**: Of those who stayed in the program for at least 365 days, 25.4% achieved their goal weight, 17.6% achieved a 10% weight loss, and 62% achieved a 5% weight loss. By 49 days, 50% had lost 2% of their weight. Significant interactions indicated that males and females in their 60s and 70s were the most responsive to the program. **Conclusions**: The online commercial Interval Weight Loss platform based on intermittent restricted eating resulted in significant weight loss in a cohort of subscribers in a real-world setting.

## 1. Introduction

Obesity continues to be a global health issue. Among adults (over the age of 18), 43% of individuals are overweight, and 16% of individuals have obesity [1]. Online and mobile weight loss platforms have been shown to be successful in promoting weight loss [2,3] and represent a cost-effective, scalable, and accessible alternative to attending in-person programs [4]. In Australia, online weight loss programs represent 29.8% of the AUD 493.3 million weight loss industry in 2024 [5].

This study outlines the weight loss results of the Interval Weight Loss program (www.intervalweightloss.com), a commercial online weight loss program based on the principle of intermittent restricted eating (IRE). IRE is a form of weight loss advice advocating alternating periods of weight loss and weight maintenance. The theory behind intermittent restricted eating focuses on avoiding the metabolic, endocrinologic, and behavioural adaptations [6] that occur with sustained energy reduction. Intermittent restricted eating is a different approach to intermittent fasting, which involves fasting or time-restricted eating, in that the IRE approach advocates periods where habitual intake is restored and alternates with periods of energy restriction.

A recent systematic review and meta-analysis found that while IRE did not result in significantly greater changes in body composition when compared with continuous energy restriction, IRE provided significant metabolic benefits in terms of preserving energy expenditure [7]. All the studies considered in the review were randomised controlled trials (RCTs) and do not represent every-day or commercial contexts. As such, this research investigates the effectiveness of IRE in a real-world setting. This study provides novel descriptive data and addresses the hypotheses that subscribers to the program for ≥365 days would achieve significant weight loss and that clinically significant weight loss would be achieved by a proportion of subscribers consistent with other weight loss interventions related in the literature.

In addition, we hypothesised that gender and age may have an effect on the weight loss achieved and that following the program would have a positive effect on behavioural change and mood.

The objectives of this research addressing the hypotheses are as follows:

1. Describe the participation patterns and dropout of subscribers to the IWL program;
2. Characterise the average long-term weight loss of subscribers;
3. Describe the success rates in terms of the proportion of subscribers who achieve their goal weight and the proportions losing 2%, 5%, and 10% of their starting weight;
4. Identify the recorded attributes associated with greater weight loss;
5. Describe the self-reported behavioural changes in response to the program.

## 2. Materials and Methods

### 2.1. The Interval Weight Loss Program

The program commenced in 2019; the theoretical framework is based on the results of the Matador study [8]. The program advocates alternating a one-month period of weight loss with a one-month period of weight maintenance. In addition, the program advocates good exercise and sleep habits. The IWL has six principles: 1. You can’t fight evolution: Impose weight loss breaks every second month to allow your body to adjust to it’s new set point. 2. Overcoming food addiction: Reach for nature first: Retrain your brain and swap processed goods for nature’s treats 3. Nutrition: The full rainbow: Eat more vegetables than you are used to and fill your plate with vegetables first. 4. Portion control: Use chopsticks: Eat big to small throughout the day and use a teaspoon, oyster fork or chopsticks with your evening meal to slow down the pace of eating. 5. Exercise: Choose to move: Incorporate incidental activity into your daily routine and focus on variety during weight loss months. 6. Better sleep: No blue light after twilight: Switch off all technology for two hours before bed and focus on hobbies such as reading, or tackle your to-do list. The IWL program focuses on improving diet, exercise, and sleep patterns.

The online IWL program offers a free trial period of 30 days. The main consideration of this analysis is long-term weight change in subscribers remaining in the program past this free period, with at least two valid weight entries. The focus is on those who remained in the program for ≥365 days. Participants were individuals who subscribed to the program of their own volition between 20 August 2019 and 5 March 2024. Data were self-reported and entered onto the online platform. The variables for this analysis included information on weight, height, BMI, goal weight, waist circumference, age, gender, and program focus. The program has two levels: IWL and IWLPlus. IWL provides access to the online material through the app and/or website with daily reminders (optout) and individualised advice based on entered data. IWLPlus provides additional access to a dietitian via unlimited email contact and in-app chat and a monthly video or phone call for an additional cost. The program is billed quarterly or annually.

A subset of participants also completed a check-in questionnaire about changes in lifestyle habits and program satisfaction.

### 2.2. Data Collection

Approval to conduct this research was received from the University of Wollongong Human Research Ethics Committee (HE2024/017). Subscribers agreed to a privacy policy allowing for their data to be used for research purposes. Data were deidentified, and participants were assigned a unique identifier prior to analysis.

### 2.3. Statistical Methods

Descriptive statistics are presented using the mean (SD) or median (IQR) as indicated. The main analysis for weight change over time was assessed using a linear mixed model with polynomial terms. AIC and BIC were used to determine the highest-order polynomial. All analysis was conducted in RStudio using R version 4.4.0 (“Puppy Cup”). Gender, age, and interaction terms for gender and time and age and time were included as covariates in the model. Survival analysis was used to determine the proportions of participants achieving 2% weight loss (percentage shown to predict success in trials as early weight loss [9]), 5% weight loss (as this weight loss has been shown to be clinically significant [10]), 10% weight loss, and the time to reach goal weight. Ten percent was chosen as this is a highly cited goal for long-term weight loss, which is reported to be met by only ~20% of people at one year [11]. Three analyses are reported for each percentage weight loss criteria: 1. Where only participants remaining in the program at that time point are considered. This is the main analysis as it answers the question about the weight loss targets at ≥365 days. This could be considered a type of on-treatment analysis. 2. The survival analysis with censoring. 3. An analysis where dropouts remain in the dataset with their last recorded weight carried forward (worst case). The differences between the base IWL program and IWLPlus were considered using a RMANOVA with an interaction term for program type and using Wilcoxon tests for within-group change and between-group differences. Due to the limited number of reported waist circumference measures, the change in waist circumference from the first to final measure was analysed using a Friedman test. An ANOVA was used to compare the difference in weight loss between the focus of the IWL principles at baseline. A McNemar’s test was used to compare the change in proportions overtime for negative and positive feelings in the check-in survey. Given that we would perform 43 tests for the primary analysis in Table 1, the interactions in Table 2, and the predictions in Table 3, an alpha level of 0.0012 was considered significant using a Bonferroni adjustment, using the standard deviation of the weight change reported from a previous study investigating weight change in an online program [12]. A total of 1705 subscribers allowed us to detect a change as low as 0.46 kg.

## 3. Results

**Figure 1:**
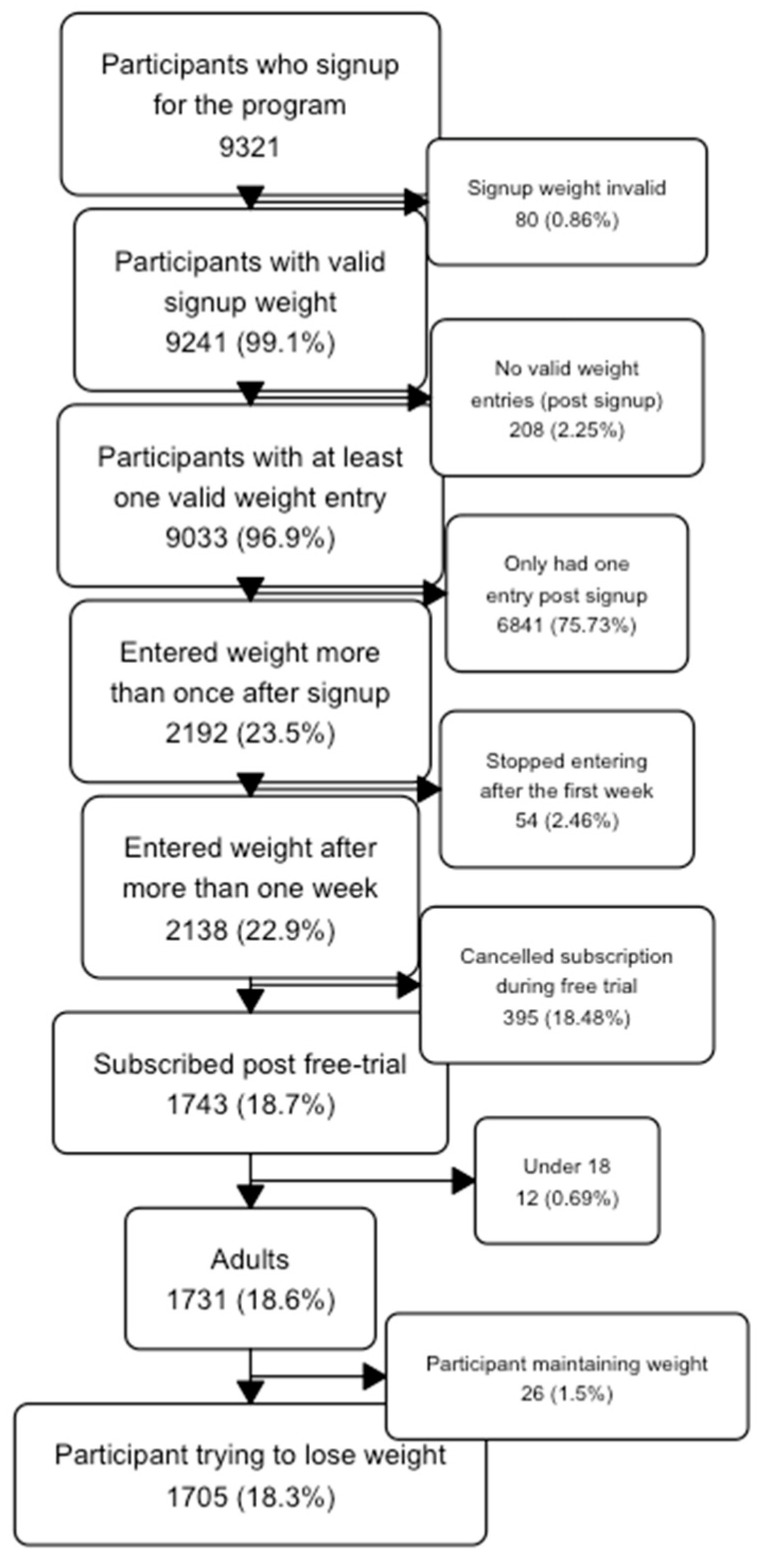
Flowchart showing participant flow.

*Participation patterns and dropout rates.* In total, 9321 participants signed up for the program with 1705 continuing to subscribe after the free trial period. A flow chart of the data selection process is shown in Figure 1. Two hundred and five participants completed the check-up satisfaction survey.

**Figure 2:**
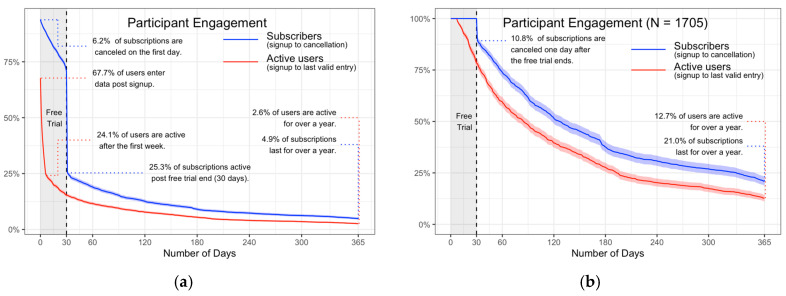
IWL program platform engagement: (**a**) platform engagement for the total dataset; (**b**) platform engagement for those subscribing past the free trial period.

Figure 2 shows the IWL program platform engagement: Figure 2a shows the platform engagement for the total dataset, and Figure 2b shows those subscribing past the free trial period. While over 2/3 of the initial subscribers entered data after signing up to the program, only 25.3% continued to subscribe after this initial trial. A subscription start date was not available for all participants; hence, full subscription data were only available for 9321 of the original participants and 1373 of those subscribing past the initial 30 days. At 12 months, 4.9% (362) of the initial participants (21.0% of those subscribing after the free trial) were still subscribing with 2.6% active (217 or 12.7% of those subscribing after the free trial).

Table 1 shows the descriptive statistics of the platform engagement of participants who subscribed past the trial period.

**Figure 3:**
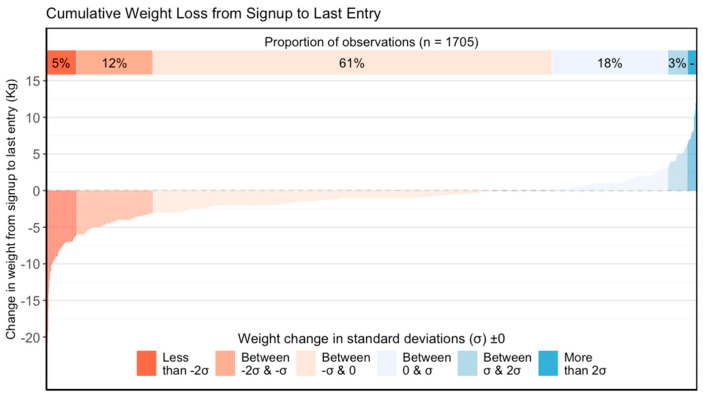
Waterfall plot showing the weight change of participants from the largest weight loss to the largest weight gain.

***Average weight loss of subscribers.*** The goal weight, weight on signup, final weight, weight change over the program, baseline and final BMI, and change in BMI are included in Table 1. CIs allow inference demonstrating an overall significant change of −1.2 kg (−1.4, −1.1); the weight change is explored further in the mixed models outlined in Table 2 and Figure 3. The change in weight in the IWLPlus subscribers (n = 324), −1.39 kg (−1.74, −1.05), was significant (*p* < 0.001), and the mean change in the IWL-only subscribers (n = 1381) was also significant, −1.17 kg (−1.33, −1.01); there was no significant difference between the two groups, 0.23 kg (95% CI −0.14, 0.59) (*p* = 0.230). Subscribers could nominate which of the IWL principles would be their initial focus in the program. Of the 784 who responded, 252 (32%) wanted to focus on portion control, 240 (31%) on food addiction, 148 (19%) on nutrition, 93 (12%) on exercise, and 51 (6%) on sleep; there was no significant difference in weight loss between these groups, F = 0.502, *df* 4, 779, *p* = 0.734. The mean change in waist circumference from baseline, 97.6 cm (95% CI 96.8, 98.5), to final measurement, 96.4 cm (95% CI 95.6, 97.2), was significant, −1.3 cm (95% CI −1.7, −0.8).

In total, 1411 participants recorded waist measurements; the median number of days active for those reporting waist measurements was 57 days (IQR 23,162), and the median number of measures was 4 days (IQR 2,10). Figure 3 details the weight change distribution in a waterfall plot: 78% of subscribers lost weight, with 61% having a loss between 3.16 kg (1 SD) and 0 kg.

**Figure 4:**
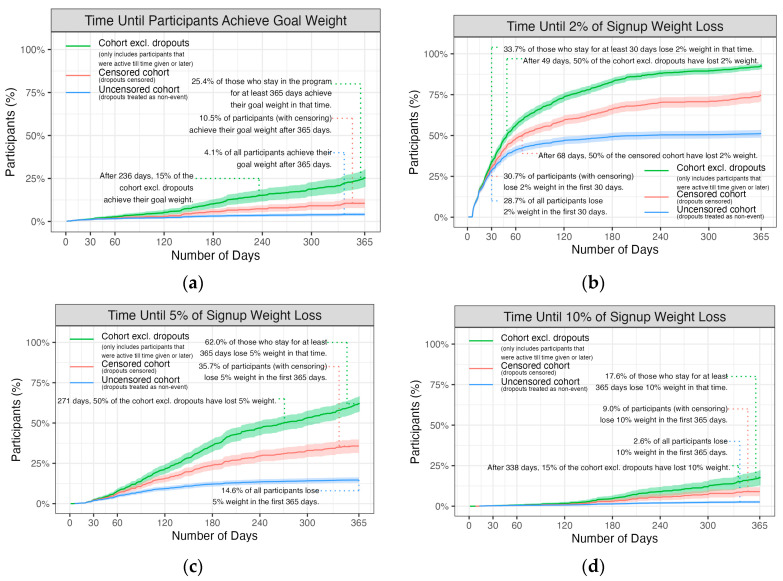
Survival analysis with dropouts censored (in orange) and the two sensitivity analyses where dropouts are excluded after their last weight entry (green) and an analysis where dropouts remain in the dataset with their last recorded weight carried forward. The analysis examined the following: (**a**) Time until participants achieve goal weight; (**b**) Time until participants achieve 2% weight loss; (**c**) Time until participants achieve 5% weight loss; (**d**) Time until participants achieve 10% weight loss. Only participants in the program after the free trial period are included.

The results of the survival analysis are presented in Figure 4. These results show the proportion of participants who achieved their goal weight and the proportion who met the 2%, 5%, and 10% weight loss criteria. The analysis was conducted on the 1705 participants who subscribed past the free trial period.

**Figure 5:**
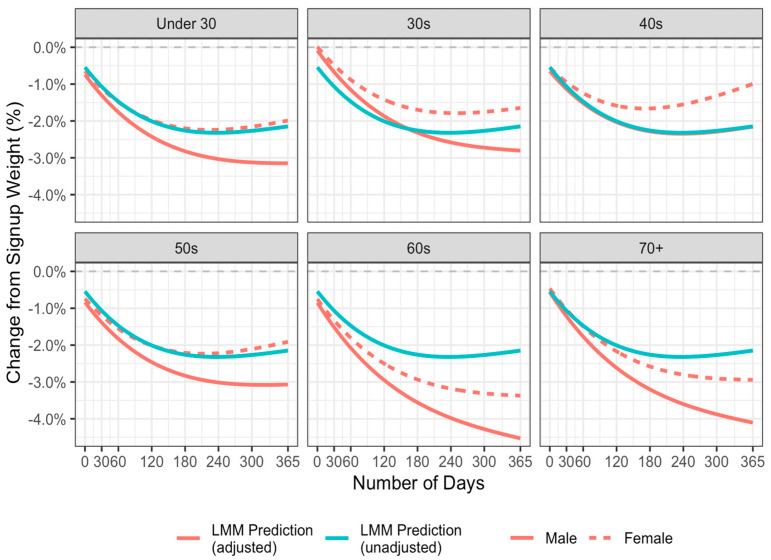
Predicted change in weight from sign-up (%) to 365 days adjusted for age and sex from the linear mixed model with polynomial regression (order = 5).

Table 2 shows the estimated coefficients, standard errors, *df*, t-values, and *p* values from the linear mixed model with polynomial regression (order = 5) adjusting for gender and age and including interaction effects with time. Figure 5 visualises these results showing both the predicted fit for the unadjusted and adjusted models. The model shows significant interactions with time and gender (with males having a greater weight loss trajectory than females) and with age, where the 40s, 60s, and 70+ categories all show significant interactions over time when compared with the reference category of under 30s. The 60s and 70s are of note as they show significantly greater trajectories of weight loss when compared with the under 30s. The significant interaction for the 40s compared with the under 30s reflects poorer outcomes for the 40s group over time when compared with the under 30s. The interaction and main effects for the 50s age group are not significantly different to the under 30s. Figure 5 shows the predicted weight loss (%) for the unadjusted and adjusted models over the 365 days. The mean predicted % weight losses at 365 days are shown in Table 3.

Table 4 and Table 5 show the results of the “check-in” survey in which a subset of subscribers completed questions on how they felt about changes they were making in the program, in line with the six principles of the IWL program, and the associated changes to their diet and lifestyle and how participating in the IWL program affected their positive and negative moods.

## 4. Discussion

This study reports on the effectiveness of the IWL online platform, a commercial weight loss program advocating an intermittent restricted eating approach, in a real-world setting. The results reported focused on 1705 users who subscribed to the program past a 30-day free trial period and presented a comprehensive summary of weight loss, dropout, and the clinical success rates of subscribers, data that are lacking in the research domain for commercial platforms. Weight loss from the first to final recorded measurement and the predicted weight loss from the mixed model at 12 months were statistically significant, averaging 1.2 kg (95% CI −1.4,−1.1). In terms of clinical impact, 62% of subscribers in the program past 365 days lost ≥5% of their body weight, a target consistently associated with improvement in health outcomes [10,13]. Significant interaction terms indicated the program was more successful for males than females and that the 60s and 70s age groups had better trajectories and the 40s age group worse when compared to the 30s age group.

The IWL diet focuses on intermittent restricted eating, a weight loss approach involving alternating periods of weight loss and weight maintenance. Sustained or continuous energy restriction results in a reduction in resting energy expenditure (REE), the major component of daily energy expenditure [14]. The reductions seen in REE are greater than what would be expected based on body composition changes, and this “adaptive thermogenesis” is thought to be a major cause of weight recidivism in long-term energy deficit. Continuous energy restriction has also been shown to be difficult to maintain long-term with high rates of attrition [15].

Intermittent fasting (IF) is another popular weight loss approach [16]. A recent systematic review comparing IF to continuous energy restriction showed a statistical but not clinically significant difference in weight and fat mass in favour of IF and significant improvements in insulin resistance in the IF trials [17]. A recent umbrella review of meta-analyses comparing RCTs of IF to control conditions showed no significant difference in weight or BMI; however, it did show substantial metabolic benefits and a reduction in body fat and waist circumference for IF over the control conditions [18]. A systematic review of RCTs showed no benefits of IF using early time-restricted feeding on energy expenditure [19]; other comparisons of the effect of IF with control diets on energy expenditure in the literature also show no difference; however, they are limited and often involve RCTs in healthy subjects [20,21].

In contrast, a systematic review and meta-analysis has recently investigated the effectiveness of intermittent restricted eating (IRE) when compared with continuous energy restriction in reducing the decline in REE in 12 randomised controlled trials ranging from 4 to 52 weeks [7]. There was a significant difference in the decline in REE between the intermittent and continuous groups, −47.29 kcal/day (−73.51, −21.07), in favour of the intermittent group (less reduction); there was no significant between-group difference in weight change (−0.01 kg 95% CI −0.70, 0.69) or BMI (0.09 kg/m^2^ 95% CI −0.31, 0.49), where a positive value favours the intermittent group. The studies evaluated were RCTs and do not extend to real-world situations nor have they specifically evaluated subscription-based online platforms. Research has shown that intermittent fasting approaches generally result in an overall energy deficit even during non-fasting periods [22,23,24]; IRE therefore represents a different approach, where caloric maintenance alternates with periods of restriction reducing the effect on energy expenditure.

Direct comparisons of intermittent fasting and intermittent energy restriction do not exist. Further investigations could clarify whether the metabolic benefits identified in conserving the reduction in energy expenditure with IRE compared with continuous energy restriction are also observed when IRE is compared with intermittent fasting. In addition, although commercial versions of the IF approach exist (for example, The fast 800), peer-reviewed evaluations of their effectiveness are lacking in a real-world context.

Many online subscription-as-service weight loss and health programs exist. Often, these focus on the composition of the diet, providing meal plans, meal-replacement products, or pre-packaged meals. Limited evaluations of the success of these commercial programs are available. One systematic review of commercial meal-replacement, calorie-counting, or pre-packaged meal RCTs and observational programs found that 57% of individuals participating lost <5% of their body weight; in total, 14 of the 25 studies were observational [25]. The programs evaluated were primarily calorie-counted meal plans (28/35 study arms), meal replacements (4/35), or pre-packaged meals. These differ to the IWL program which advocates whole lifestyle changes in food, exercise, and sleep in the context of IRE. Despite the different approaches, the proportion achieving >5% weight loss was similar (62% in the present study compared with 63% in the meta-analysis) for completers and (35.7% compared with 41%) in the intention to treat/censored analysis, noting that 67% of the studies were ≤24 weeks, meaning success rates are likely to be higher as weight loss is most rapid during this period [26]. These figures (62% and 35.7%) are substantially higher than the 10% probability of achieving a >5% weight loss over a year calculated from a large US-population-based overweight cohort [27].

More recent literature reporting on the effectiveness of online weight loss programs in the real world include The CSIRO Total Wellbeing Diet Online [12], a 12-week intervention period followed by a maintenance period evaluated at the 24-week mark reporting an overall average weight loss of 2.84 kg (SD 4.69). The analysis is divided into stayers who completed the initial 12-week program and starters who did not complete this component. In total, 48.9% of stayers and 9.6% of starters lost ≥5% of their starting weight, with a mean overall weight loss of 2.84 (4.69)kg. The Noom Weight online program [28] has been evaluated retrospectively in an observational cross-sectional study reporting on participants who had unsubscribed but had previously completed the 16-week core program and lost at least 10% of their weight; this study reported that 75% maintained ≥5% weight loss at 12 months. This cohort differs in that it only considers those that had lost 10% in the program, resulting in a sample biased in being successful. It is mentioned here as it is one of the few [28] studies that mentions a whole lifestyle approach focusing not only on dietary changes but on behavioural changes in relation to exercise using cognitive behavioural therapy, acceptance and commitment therapy, and dialectical behavioural therapy. The WeightWatchers (WW) program has been shown to be effective over 12 months in randomized controlled trials [29]; while there are no studies of the online program in an everyday setting, a single-arm behavioural trial over 6 months is one of the few other studies reporting on satisfaction outcomes and quality of life, using a composite well-being index scale incorporating similar components to those shown in Table 5. The WW online program was also shown to improve positive affect [30]. Early weight loss (2%) has been shown to be important in predicting success in weight loss trials [9]; in this cohort, we found that 33.3% of those staying in the program past 30 days lost ≥2% of their starting weight. As weight losses less than this also predict dropout in clinical trials [31], it is worth investigating whether the targeted follow-up of subscribers failing to meet this early predicted value could reduce dropout in the real-world context.

The clinical benefits of weight loss for multiple health outcomes, including glycaemic improvement in controlling diabetes; triglyceride reduction; improvement in functionality in osteoarthritis; reductions in knee pain; improvements in quality of life, urinary incontinence, and sexual function; and reduction in hospitalisations, all start to occur with reductions in weight in the 5–10% range [13]. Maintaining ≥10% weight loss is widely reported as a goal for the reduction of multiple comorbidities including diabetes and cardiovascular disease, improving quality of life [11,13,32] and reducing mortality [13]. Maintaining this weight loss has been shown to be difficult to achieve. Results achieved in the IWL cohort found 17.6% of those staying on the program over 365 days reported achieving a 10% weight loss, a figure higher than the 15.4% reported for the CSIRO Total Wellbeing Diet [12] and the 14.4% in a single-arm evaluation of the digital WeightWatchers program [30]. Importantly, 25.4% of participants met their goal weight; this is notable given that it has been shown that people with obesity have unrealistic expectations about the amount of weight they can lose [33], and it is a percentage higher than found in the literature [34].

Online weight loss programs represented 29.8% of the AUD 493.3 million weight loss market in Australia in 2024 [5]. Similarly, market data from the US shows the weight loss industry there was at its highest level in 2023, with an estimated worth of USD 90 billion, having increased with the introduction of new weight loss drugs; this growth is anticipated to continue as new drugs become available. Other components of the industry such as commercial online weight loss programs have been shown to be decreasing in demand unless this medical component was incorporated. It is suggested that along with a move towards medical treatments (such as GLP-1 receptor agonists), the decrease in discretionary spending may be contributing to a reduction in subscriptions. A systematic review of the RCTs of GLP-1 receptor agonists showed 50.2% of those taking active treatment in the RCTs had a weight loss ≥5% at one year [35]. A more recent study investigating the one-year effectiveness of Semaglutide and Liraglutide in clinical practice found proportions of 55.4% and 37% [36], respectively, (intention to treat) and 81.6% and 52.4% (on treatment) having lost ≥5% at one year; both figures are in the range of the 35.7% with censoring or the 62% on treatment having ≥5% in the present study. Although these figures show that the medical management of obesity may have higher success rates for some medications, the adverse events, cost, and availability issues associated with these treatments and the focus on long-term behaviour modification suggest there is still a long-term role for alternative weight loss treatments such as online weight loss platforms.

Both the medical and surgical management of obesity are associated with adverse events and do not inherently cause behavioural change. eHealth and online weight loss interventions have obvious advantages in terms of scalability and ease of access when compared with face-to-face interventions. A recent systematic review of systematic reviews [3] showed that while the difference between using eHealth applications and face-to-face interventions was not significant, 0.12 kg (−0.64, 0.41), they did show a significant effect when compared to no care, −4.23 kg (−5.98, −3.57). Mobile applications have also been shown to be effective in promoting weight loss when compared with traditional interventions, with a meta-analysis of 12 studies showing a reduction of −1.07 kg, 95% CI −1.92 to −0.21, *p* = 0.01, compared to control interventions [4].

The results in this study show strong gender and age effects on the weight loss achieved. The gender effect is consistent with previous research on RCTs of weight loss where males have been shown to respond better [37]. This may represent participation bias as the proportion of males participating is lower, and they may be a potentially more motivated group, for example, Robertson et al. (2014) [38] in a systematic review found men were less likely to drop out of weight loss trials, suggesting higher motivation. Women are more likely to have tried previous diets than men [39], and this may result in poor metabolic outcomes [40] and may mean women are less likely to respond to the intervention. The age effect is also one observed previously, in our research [31] and others’ [37], as older participants may have less time pressure going into retirement and more time to focus on the program [41,42], as opposed to those in their 40s potentially in the peak of childrearing and career activities.

The check-in survey completed by a subset of subscribers demonstrated that dietary-related behavioural changes were perceived as the most substantial changes made, and lifestyle-related changes around exercise, technology, sleep, and organization were the ones that still needed to be worked on. Overall, subscribing to the program resulted in a significantly greater proportion of participants feeling balanced, calm, mostly happy, and optimistic since commencing the IWL program and significant reductions in the proportion of feeling anxious, busy, depressed, mostly unhappy, and stressed. This study provides unique data on platform engagement and attrition over a one-year period. Hendrie et al. [12] reported on platform attrition over 24 weeks for paid subscribers in the CSIRO Total Wellbeing Diet, with 26.59% of subscribers remaining active users at 24 weeks. While the dietary approach in the study was different to the IWL program in this study in that the Total Wellbeing Diet is a continuous energy restriction diet for 12 weeks followed by weight maintenance, it is one of the few commercial programs reporting on platform engagement. Our analysis showed that of the 1705 remaining in the program after the free trial period, 21% were still subscribing and 12.7% were still active with weigh-ins after one year, noting that many of the 228 commencing the program in 2023 and the 58 commencing in 2024 could not have been part of this proportion. The total number of days of platform engagement for weigh-in on the Total Wellbeing Diet was 12.27 days (SD 24.09), similar to the present study where the average was 13 days (95% CI 12,14). The number of days active was higher on the IWL program, median 86 days (IQR 39,193), mean 174 days (95% CI 163,185), compared with the Total Wellbeing Diet, mean 48.17 days (79.86). The active days variable was right skewed, and further research could investigate the factors associated with platform engagement, particularly at the higher levels. Brankovic et al. (2023) [43] used machine learning on the Total Wellbeing Diet dataset to examine the factors predicting disengagement with the platform and found that weigh-ins the week before and total website activity were the best predictors. Behavioural factors including skipping meals and higher levels of emotional eating also predicted platform disengagement. It would be useful if characteristics recorded or reported at the time of subscription could be identified to allow the development of early targeted approaches.

We report novel data on the dropout rates in the free trial period. While many other commercial online weight loss programs offer a free trial or discounted periods (e.g., The fast 800, WW WeightWatchers^®^ online, Ultimo, NSW, Australia), there is limited published data on the take-up rates of the program after this period. Our results demonstrate that 81.3% drop out after the free trial period. Given that this is a commercial program, predicting churn or user dropout is useful for developing strategies for retention and better commercial success. A previous systematic review reporting on the factors that improved adherence (the ratio between intended and actual use) as markers for retention in nine weight loss apps found the mean to be 49.1%, with positive components including push notification reminders, just in time (adaptive) intervention components, newsfeeds with social aspects, personal contact, and interaction with health care professionals. Rural populations, positive expectations, a sense of responsibility, and reinforcements were positively related to adherence [44]. Jakob et al. [45] investigated early churn users on a digital weight loss intervention and found 65% dropped out in a free 7-day trial period or within the 14 days following this. Their analysis showed early platform engagement was the best predictor; however, user weight was the only non-platform-based attribute considered. It would be interesting to investigate the effects of these promotions on the engagement and success of the programs given that long-term weight loss and maintenance is the goal. Further investigation of participant-related features such as age; educational, socioeconomic, and other demographic factors; quality of life; baseline mood; and other psychosocial factors could also provide information about subscriber adherence to online programs. For example, it has previously been found that symptoms of depression are associated with reduced adherence in one weight loss app [46]. Churn prevention measures in the IWL program included opt in daily reminder notifications, an email offering an additional support call with an IWL dietitian, and points for achieving self-set goals; data were not available to assess the effectiveness of these messages. Predicting churn from commercial programs and implementing strategies to counteract this would result in better health and commercial outcomes.

### Limitations

The main aim of this study was to examine the success of the long-term weight loss (≥365 days) of subscribers to the IWL program. A substantial number of participants enrolled in the program in 2023 (n = 412) and some in early 2024 (n = 84); as the analysis cut-off was in March 2024, many of these participants would not have been able to provide data for periods in excess of 12 months, even if they continued to subscribe and remained active. Only 12% of the subscribers completed the check-in survey, and their view may differ from the rest of the subscriber base. Further research could involve a longer time frame for evaluation and a widening of the subscriber base completing the check-up survey. In addition, including validated surveys of other lifestyle-related factors such as dietary intake, sleep quality, physical activity, or psychological well-being could provide insights into program success and attrition and allow the development of predictive models. The participant burden of completing this in a commercial setting would have to be considered.

All data included are based on the self-reporting of subscribers; as this was a real-world evaluation, validation studies on subsets of the participants were not conducted. This issue is not unique to our study, and previous research suggests that while there is some bias in the self-reporting of web-based weight data, it can be a valid method. In addition, the correlation between the reported and measured data was high (r = 0.98) [47]. As our primary interest was the change in weight, it is anticipated that any bias would be consistent over time and that the change in weight would be accurate. This is also untested in the current evaluation and remains a limitation of this study.

As the online platform was not specifically designed to collect data for research purposes, standard demographic data such as socioeconomic factors and educational levels were not requested and therefore could not be considered in this analysis. This also meant we were unable to assess the generalizability of the program to different populations as information on the location and background of the subscribers was not available. Again, being mindful of the commercial context and participant burden in collecting this information, for future analysis purposes, such data would allow for comparisons with a broader population.

As this was a retrospective assessment of a commercial program and not a prospective controlled intervention, it is not possible to attribute the weight loss specifically to the IWL program nor was it possible to blind the participants to the intervention; this should be noted when interpreting the results of this study.

## 5. Conclusions

This research is the first to report on the use of intermittent restricted eating in a commercial weight loss program, the Interval Weight Loss program, in a real-world setting. The results demonstrate a clinically meaningful weight loss in 62% of those staying in the program for at least 365 days. Interaction terms indicate better responses for males and those in their 60s and 70s. Attrition rates were high; however, they were similar to those in the existing literature. Participants reported positive mood changes with the program. Further research could expand the study period and include additional demographic and lifestyle measures to gain deeper insights into the factors influencing success and subscriber retention. Furthermore, future studies could explore whether intermittent restricted eating has the metabolic benefits demonstrated in clinical trials when applied in the real-world context.

## Figures and Tables

**Table 1:** Descriptive statistics outlining subscription length and activity, goal weight, weight change, and BMI change.

n Mean (CI) Median (IQR)	Subscription Length	Number of Days Active	Number of Weight Entries	Average Days Between Entries	Goal Weight (kg)	Weight (kg) on Signup	Weight (kg) on Final Entry	Change in Weight over Program	BMI on Signup	BMI on Final Entry	Change in BMI over Program
Total	n = 1373 215 (203, 227) 127 (58, 333)	n = 1705 174 (163, 185) 85 (35, 193)	n = 1705 13 (12, 14) 7 (3, 16)	n = 1705 16 (15, 17) 9 (7, 14)	n = 1704 75.0 (74.3, 75.7) 72.9 (64.8, 82.0)	n = 1705 85.5 (84.7, 86.4) 83.0 (72.3, 95.2)	n = 1705 84.3 (83.5, 85.2) 82.0 (71.0, 94.0)	**n = 1705** **−1.2 (−1.4, −1.1)** **−1.0 (−2.2, 0.0)**	n = 1578 30.9 (30.6, 31.2) 30.0 (26.7, 33.9)	n = 1578 30.4 (30.1, 30.7) 29.4 (26.4, 33.6)	**n = 1578** **−0.4 (−0.5, −0.4)** **−0.4 (−0.8, 0.0)**
**By Gender** Female	n = 1192 218 (205, 231) 130 (59, 337)	n = 1463 178 (165, 190) 88 (35, 196)	n = 1463 13 (12, 14) 7 (3, 16)	n = 1463 17 (15, 18) 10 (7, 14)	n = 1462 72.5 (71.9, 73.1) 71.0 (63.9, 79.1)	n = 1463 83.1 (82.3, 84.0) 80.9 (71.1, 91.8)	n = 1463 82.0 (81.2, 82.9) 80.0 (70.0, 91.0)	**n = 1463** **−1.1 (−1.3, −1.0)** **−1.0 (−2.0, 0.0)**	n = 1354 30.7 (30.4, 31.0) 29.8 (26.5, 33.6)	n = 1354 30.3 (30.0, 30.6) 29.2 (26.3, 33.5)	**n = 1354** **−0.4 (−0.5, −0.4)** **−0.4 (−0.8, 0.0)**
Male	n = 181 198 (166, 230) 120 (56, 246)	n = 242 152 (124, 179) 74 (34, 182)	n = 242 13 (11, 16) 7 (3, 15)	n = 242 13 (11, 14) 9 (7, 14)	n = 242 90.1 (88.0, 92.2) 88.4 (79.2, 99.3)	n = 242 100.1 (97.6, 102.6) 98.8 (85.6, 113.7)	n = 242 98.2 (95.7, 100.8) 95.0 (84.0, 111.8)	**n = 242** **−1.8 (−2.3, −1.4)** **−1.8 (−3.0, 0.0)**	n = 224 31.7 (30.9, 32.5) 31.3 (27.8, 35.1)	n = 224 31.1 (30.3, 31.9) 30.2 (27.0, 34.4)	**n = 224** **−0.6 (−0.8, −0.4)** **−0.5 (−1.1, 0.0)**
**By Age Group**											
Under 30	n = 50 203 (124, 281) 94 (57, 181)	n = 56 182 (101, 264) 55 (29, 116)	n = 56 12 (6, 18) 5 (3, 10)	n = 56 20 (9, 31) 10 (7, 16)	n = 56 74.8 (71.0, 78.6) 74.0 (62.2, 83.9)	n = 56 82.8 (78.0, 87.6) 80.2 (70.0, 94.5)	n = 56 81.9 (77.2, 86.6) 82.0 (68.0, 93.0)	**n = 56** **−0.9 (−1.6, −0.2)** **−0.8 (−2.0, 0.4)**	n = 53 29.1 (27.4, 30.7) 28.0 (25.2, 31.9)	n = 53 28.8 (27.2, 30.4) 28.1 (24.6, 31.6)	**n = 53** **−0.3 (−0.6, −0.0)** **−0.2 (−0.7, 0.3)**
30s	n = 209 209 (180, 237) 131 (62, 322)	n = 251 168 (139, 197) 81 (36, 181)	n = 251 12 (10, 14) 7 (4, 13)	n = 251 16 (14, 18) 10 (8, 16)	n = 251 77.8 (75.8, 79.9) 74.8 (66.5, 87.2)	n = 251 88.0 (85.5, 90.5) 84.0 (74.5, 98.6)	n = 251 87.2 (84.7, 89.7) 83.0 (73.0, 98.0)	**n = 251** **−0.8 (−1.2, −0.4)** **−0.8 (−2.0, 0.8)**	n = 234 30.9 (30.1, 31.6) 29.8 (26.6, 34.1)	n = 234 30.6 (29.8, 31.3) 29.3 (26.2, 34.5)	**n = 234** **−0.3 (−0.4, −0.1)** **−0.3 (−0.7, 0.3)**
40s	n = 379 220 (200, 241) 152 (70, 348)	n = 452 171 (151, 191) 92 (35, 200)	n = 452 13 (11, 15) 7 (4, 15)	n = 452 17 (15, 20) 10 (7, 15)	n = 451 75.3 (74.0, 76.7) 73.0 (65.5, 82.0)	n = 452 86.0 (84.4, 87.7) 84.0 (73.0, 96.0)	n = 452 85.2 (83.5, 86.8) 82.0 (72.0, 95.0)	**n = 452** **−0.9 (−1.2, −0.6)** **−1.0 (−2.0, 0.0)**	n = 425 31.0 (30.4, 31.5) 30.3 (26.6, 33.9)	n = 425 30.6 (30.1, 31.2) 29.8 (26.5, 33.7)	**n = 425** **−0.3 (−0.4, −0.2)** **−0.3 (−0.8, 0.0)**
50s	n = 421 220 (197, 243) 120 (54, 332)	n = 524 169 (149, 190) 77 (32, 194)	n = 524 13 (11, 14) 6 (3, 14)	n = 524 17 (15, 20) 10 (7, 14)	n = 524 74.4 (73.3, 75.6) 72.7 (64.5, 82.2)	n = 524 85.5 (83.9, 87.0) 82.1 (71.5, 96.0)	n = 524 84.2 (82.6, 85.7) 82.0 (70.8, 94.0)	**n = 524** **−1.3 (−1.6, −1.1)** **−1.0 (−2.1, 0.0)**	n = 486 31.0 (30.5, 31.5) 30.1 (26.7, 34.1)	n = 486 30.5 (30.0, 31.1) 29.4 (26.3, 33.7)	**n = 486** **−0.5 (−0.6, −0.4)** **−0.4 (−0.8, 0.0)**
60s	n = 270 208 (180, 236) 108 (52, 313)	n = 359 186 (158, 213) 88 (40, 194)	n = 359 15 (13, 17) 8 (4, 20)	n = 359 13 (11, 15) 9 (7, 12)	n = 359 73.0 (71.6, 74.4) 70.5 (64.0, 80.0)	n = 359 83.4 (81.6, 85.1) 81.0 (72.2, 91.0)	n = 359 81.6 (79.8, 83.3) 80.0 (71.0, 89.5)	**n = 359** **−1.8 (−2.1, −1.5)** **−1.3 (−3.0, −0.3)**	n = 323 30.7 (30.1, 31.3) 29.8 (26.9, 33.4)	n = 323 30.0 (29.5, 30.6) 29.0 (26.4, 33.1)	**n = 323** **−0.6 (−0.8, −0.5)** **−0.5 (−1.1, −0.1)**
70+	n = 44 214 (139, 289) 119 (54, 313)	n = 63 182 (128, 236) 108 (42, 207)	n = 63 15 (11, 18) 10 (5, 20)	n = 63 13 (10, 16) 8 (7, 13)	n = 63 77.5 (73.9, 81.1) 76.2 (69.1, 80.2)	n = 63 87.4 (83.4, 91.4) 86.5 (79.2, 94.5)	n = 63 85.8 (81.8, 89.9) 86.0 (77.0, 92.5)	**n = 63** **−1.6 (−2.2, −1.0)** **−1.0 (−2.4, 0.0)**	n = 57 31.8 (30.5, 33.0) 31.8 (28.5, 35.1)	n = 57 31.3 (30.0, 32.5) 30.5 (28.1, 34.5)	**n = 57** **−0.5 (−0.8, −0.3)** **−0.4 (−0.8, 0.0)**
**BMI** Healthy Weight	n = 151 211 (177, 246) 116 (56, 294)	n = 185 179 (143, 215) 84 (32, 183)	n = 185 13 (10, 15) 6 (3, 15)	n = 185 19 (14, 24) 10 (7, 15)	n = 185 60.6 (59.7, 61.6) 60.0 (56.0, 64.0)	n = 185 65.4 (64.4, 66.4) 65.0 (60.0, 69.2)	n = 185 64.6 (63.6, 65.7) 64.0 (59.0, 69.0)	**n = 185** **−0.8 (−1.2, −0.4)** **−1.0 (−2.0, 0.0)**	n = 185 23.5 (23.4, 23.7) 23.7 (23.0, 24.5)	n = 185 23.2 (23.0, 23.5) 23.3 (22.3, 24.2)	**n = 185** **−0.3 (−0.4, −0.2)** **−0.3 (−0.7, 0.0)**
Overweight	n = 479 202 (182, 221) 120 (56, 288)	n = 602 172 (152, 191) 86 (34, 192)	n = 602 14 (12, 15) 7 (4, 17)	n = 602 15 (13, 17) 9 (7, 13)	n = 602 68.8 (68.1, 69.4) 67.8 (62.8, 73.2)	n = 602 76.3 (75.6, 77.0) 75.4 (70.0, 81.3)	n = 602 75.2 (74.5, 75.9) 74.0 (69.0, 81.0)	**n = 602** **−1.1 (−1.3, −0.9)** **−1.0 (−2.0, 0.0)**	n = 602 27.5 (27.4, 27.6) 27.6 (26.2, 28.8)	n = 602 27.1 (27.0, 27.2) 27.1 (25.9, 28.4)	**n = 602** **−0.4 (−0.5, −0.3)** **−0.4 (−0.8, 0.0)**
Obese Class I	n = 367 216 (193, 239) 133 (54, 351)	n = 469 164 (144, 184) 88 (34, 196)	n = 469 13 (11, 14) 7 (3, 15)	n = 469 15 (14, 17) 10 (7, 14)	n = 469 78.2 (77.3, 79.0) 76.6 (71.6, 83.1)	n = 469 89.9 (89.0, 90.8) 89.0 (83.0, 95.9)	n = 469 88.4 (87.5, 89.4) 88.0 (81.0, 95.0)	**n = 469** **−1.4 (−1.7, −1.1)** **−1.0 (−2.6, 0.0)**	n = 469 32.2 (32.1, 32.3) 32.1 (31.1, 33.3)	n = 469 31.7 (31.5, 31.9) 31.6 (30.4, 33.1)	**n = 469** **−0.5 (−0.6, −0.4)** **−0.4 (−0.9, 0.0)**
Obese Class II	n = 171 224 (191, 258) 130 (68, 338)	n = 215 169 (142, 197) 78 (40, 206)	n = 215 14 (11, 16) 7 (3, 15)	n = 215 16 (13, 18) 10 (7, 16)	n = 214 87.3 (85.7, 88.9) 85.4 (79.5, 92.0)	n = 215 103.2 (101.7, 104.7) 101.0 (95.5, 109.2)	n = 215 101.9 (100.4, 103.4) 101.0 (94.0, 109.0)	**n = 215** **−1.3 (−1.7, −0.9)** **−1.0 (−2.5, 0.0)**	n = 215 36.8 (36.6, 37.0) 36.6 (35.6, 37.7)	n = 215 36.3 (36.1, 36.6) 36.3 (35.2, 37.5)	**n = 215** **−0.5 (−0.6, −0.3)** **−0.4 (−0.9, 0.0)**
Obese Class III	n = 87 244 (186, 302) 124 (58, 356)	n = 107 182 (137, 228) 75 (30, 258)	n = 107 12 (9, 15) 6 (3, 14)	n = 107 19 (14, 25) 10 (7, 17)	n = 107 101.0 (97.8, 104.2) 97.5 (89.2, 110.5)	n = 107 123.1 (119.6, 126.6) 121.2 (110.0, 130.6)	n = 107 121.4 (117.7, 125.1) 118.0 (109.0, 129.0)	**n = 107** **−1.7 (−2.5, −0.9)** **−1.0 (−3.0, 0.0)**	n = 107 44.7 (43.7, 45.6) 43.2 (40.9, 47.0)	n = 107 44.0 (43.1, 45.0) 42.3 (40.5, 46.4)	**n = 107** **−0.6 (−0.9, −0.3)** **−0.4 (−1.2, 0.0)**
**Year** 2019	n = 40 545 (426, 664) 520 (263, 648)	n = 52 538 (415, 662) 374 (250, 693)	n = 52 35 (26, 44) 24 (11, 50)	n = 52 24 (15, 33) 15 (11, 23)	n = 51 74.9 (70.9, 78.9) 73.0 (65.0, 82.8)	n = 52 86.0 (80.9, 91.0) 84.7 (72.2, 97.8)	n = 52 85.1 (80.1, 90.1) 85.0 (72.8, 95.2)	**n = 52** **−0.8 (−2.3, 0.6)** **0.0 (−2.1, 1.2)**	n = 49 31.0 (29.4, 32.6) 31.6 (26.5, 35.6)	n = 49 30.8 (29.1, 32.5) 31.2 (25.9, 34.4)	**n = 49** **−0.2 (−0.7, 0.3)** **0.0 (−0.8, 0.5)**
2020	n = 630 213 (194, 232) 130 (62, 235)	n = 673 181 (161, 202) 75 (36, 174)	n = 673 14 (12, 16) 7 (4, 15)	n = 673 17 (14, 19) 9 (7, 14)	n = 673 74.5 (73.4, 75.7) 72.3 (63.8, 82.0)	n = 673 85.1 (83.7, 86.5) 82.2 (71.2, 95.0)	n = 673 84.1 (82.7, 85.5) 82.0 (70.0, 94.0)	**n = 673** **−1.0 (−1.2, −0.8)** **−1.0 (−2.0, 0.0)**	n = 578 30.9 (30.4, 31.4) 30.0 (26.8, 33.7)	n = 578 30.6 (30.1, 31.0) 29.5 (26.5, 33.6)	**n = 578** **−0.4 (−0.4, −0.3)** **−0.3 (−0.8, 0.0)**
2021	n = 260 277 (251, 303) 234 (92, 366)	n = 294 205 (178, 232) 109 (42, 301)	n = 294 14 (12, 16) 8 (4, 17)	n = 294 18 (15, 21) 11 (8, 16)	n = 294 75.2 (73.6, 76.7) 72.5 (66.1, 81.0)	n = 294 85.7 (83.6, 87.7) 82.8 (73.2, 94.2)	n = 294 84.4 (82.3, 86.4) 81.0 (72.0, 93.0)	**n = 294** **−1.3 (−1.7, −0.9)** **−1.0 (−3.0, 0.0)**	n = 266 30.5 (29.8, 31.2) 29.5 (26.3, 33.3)	n = 266 30.1 (29.4, 30.8) 29.0 (26.0, 33.3)	**n = 266** **−0.5 (−0.6, −0.3)** **−0.4 (−1.0, 0.0)**
2022	n = 157 280 (253, 308) 308 (117, 380)	n = 190 203 (175, 231) 133 (48, 342)	n = 190 14 (12, 16) 7 (3, 17)	n = 190 20 (17, 24) 12 (9, 21)	n = 190 75.1 (73.1, 77.2) 73.2 (65.0, 81.8)	n = 190 86.0 (83.3, 88.6) 82.0 (72.2, 98.8)	n = 190 84.3 (81.6, 87.0) 81.0 (71.2, 94.8)	**n = 190** **−1.7 (−2.1, −1.3)** **−1.0 (−3.0, 0.0)**	n = 190 31.0 (30.1, 31.8) 29.7 (26.4, 35.4)	n = 190 30.4 (29.5, 31.2) 28.8 (25.8, 34.1)	**n = 190** **−0.6 (−0.8, −0.5)** **−0.4 (−1.1, 0.0)**
2023	n = 228 95 (84, 107) 65 (31, 120)	n = 412 109 (101, 118) 86 (29, 171)	n = 412 11 (10, 11) 8 (3, 15)	n = 412 12 (11, 14) 8 (7, 12)	n = 412 75.9 (74.5, 77.2) 73.4 (66.1, 83.6)	n = 412 86.2 (84.6, 87.9) 83.9 (74.0, 96.0)	n = 412 84.9 (83.2, 86.6) 82.0 (72.0, 95.0)	**n = 412** **−1.3 (−1.6, −1.1)** **−1.0 (−2.3, 0.0)**	n = 411 31.0 (30.5, 31.5) 30.1 (27.2, 33.9)	n = 411 30.5 (30.0, 31.1) 29.4 (26.9, 33.5)	**n = 411** **−0.5 (−0.6, −0.4)** **−0.4 (−0.9, 0.0)**
2024	n = 58 31 (31, 32) 31 (31, 31)	n = 84 29 (26, 32) 26 (16, 42)	n = 84 4 (4, 4) 4 (2, 5)	n = 84 7 (7, 8) 7 (6, 8)	n = 84 73.5 (71.0, 76.1) 72.8 (64.8, 81.0)	n = 84 83.8 (80.6, 86.9) 82.0 (72.0, 94.2)	n = 84 82.5 (79.3, 85.7) 81.0 (72.0, 93.0)	**n = 84** **−1.3 (−1.5, −1.0)** **−1.1 (−2.0, −0.6)**	n = 84 30.5 (29.5, 31.5) 30.2 (26.7, 33.2)	n = 84 30.0 (29.0, 31.0) 29.9 (26.6, 32.4)	**n = 84** **−0.5 (−0.6, −0.4)** **−0.4 (−0.7, −0.2)**
**Time in Program** First 100 days	n = 848 116 (108, 125) 71 (41, 121)	n = 940 44 (42, 45) 39 (23, 62)	n = 940 5 (5, 5) 4 (3, 7)	n = 940 10 (9, 10) 8 (6, 10)	n = 940 75.4 (74.4, 76.3) 73.0 (64.8, 83.4)	n = 940 85.6 (84.5, 86.8) 83.0 (72.0, 96.0)	n = 940 84.7 (83.5, 85.9) 82.0 (71.0, 95.0)	**n = 940** **−0.9 (−1.1, −0.8)** **−1.0 (−2.0, 0.0)**	n = 866 30.8 (30.5, 31.2) 30.0 (26.7, 33.7)	n = 866 30.5 (30.1, 30.9) 29.7 (26.4, 33.6)	**n = 866** **−0.3 (−0.4, −0.3)** **−0.4 (−0.7, 0.0)**
100–200 days	n = 250 213 (199, 226) 176 (142, 241)	n = 364 149 (146, 152) 148 (120, 174)	n = 364 13 (13, 14) 13 (8, 18)	n = 364 16 (15, 18) 10 (8, 16)	n = 364 74.5 (73.0, 75.9) 72.5 (64.7, 81.0)	n = 364 84.8 (83.1, 86.6) 82.3 (73.0, 93.4)	n = 364 83.4 (81.6, 85.1) 80.0 (72.0, 93.0)	**n = 364** **−1.5 (−1.8, −1.2)** **−1.5 (−3.0, 0.0)**	n = 341 30.6 (30.0, 31.1) 29.8 (26.6, 33.9)	n = 341 30.1 (29.5, 30.6) 29.0 (26.2, 33.5)	**n = 341** **−0.5 (−0.6, −0.4)** **−0.5 (−1.1, 0.0)**
200 days–1 year	n = 141 361 (336, 386) 355 (269, 374)	n = 184 284 (276, 291) 292 (234, 336)	n = 184 21 (20, 23) 22 (13, 28)	n = 184 22 (18, 26) 13 (10, 21)	n = 184 75.6 (73.8, 77.5) 74.3 (67.3, 81.0)	n = 184 86.8 (84.3, 89.4) 84.0 (74.3, 94.4)	n = 184 85.0 (82.4, 87.5) 81.5 (73.0, 94.0)	**n = 184** **−1.9 (−2.5, −1.3)** **−1.3 (−4.3, 0.1)**	n = 176 31.2 (30.4, 32.1) 30.1 (27.3, 33.7)	n = 176 30.5 (29.7, 31.4) 29.1 (26.7, 33.4)	**n = 176** **−0.7 (−0.9, −0.5)** **−0.5 (−1.5, 0.0)**
Over a year	n = 134 693 (649, 738) 648 (480, 805)	n = 217 687 (646, 727) 581 (463, 804)	n = 217 43 (38, 47) 40 (17, 57)	n = 217 39 (31, 46) 16 (10, 36)	n = 216 73.8 (71.9, 75.8) 71.0 (62.8, 81.4)	n = 217 85.1 (82.5, 87.8) 81.0 (70.5, 97.0)	n = 217 83.7 (81.1, 86.4) 80.0 (70.0, 93.0)	**n = 217** **−1.4 (−2.1, −0.7)** **−1.0 (−3.9, 1.0)**	n = 195 31.2 (30.3, 32.2) 30.2 (26.2, 34.8)	n = 195 30.7 (29.8, 31.7) 29.2 (26.2, 34.2)	**n = 195** **−0.5 (−0.8, −0.3)** **−0.4 (−1.4, 0.4)**

**Table 2:** Coefficient estimates (standard errors), degrees of freedom, t values, and significance results from the mixed model.

Term	Estimate	Std. Error	*df*	t Value	*p*
Intercept	−1.604	0.320	2026	−5.02	<0.001 *
Time (in weeks) order = 1	−10.139	11.718	22,304	−0.87	0.387
Time (in weeks) order = 2	47.049	2.604	22,369	18.07	<0.001 *
Time (in weeks) order = 3	−27.776	2.385	22,119	−11.65	<0.001 *
Time (in weeks) order = 4	49.248	2.330	22,005	21.14	<0.001 *
Time (in weeks) order = 5	−10.725	2.270	21,875	−4.72	<0.001 *
Male − Female	−0.099	0.174	1954	−0.57	0.568
30s − Under 30	0.659	0.355	2034	1.85	0.064
40s − Under 30	0.097	0.340	2041	0.29	0.775
50s − Under 30	−0.094	0.337	2047	−0.28	0.781
60s − Under 30	−0.104	0.344	2024	−0.30	0.762
70+ − Under 30	0.181	0.445	1954	0.41	0.685
Male × Time (in weeks)	−0.020	0.002	22,289	−11.76	<0.001 *
30s × Time (in weeks)	−0.006	0.002	22,272	−2.62	0.009
40s × Time (in weeks)	0.017	0.002	22,282	7.65	<0.001 *
50s × Time (in weeks)	0.003	0.002	22,381	1.49	0.136
60s × Time (in weeks)	−0.025	0.002	22,281	−11.29	<0.001 *
70+ × Time (in weeks)	−0.022	0.004	22,408	−6.09	<0.001 *

* indicates statistical significance at *p* < 0.0012, × refers to an interaction term in the model.

**Table 3:** Predicted weight changes (%) at 365 days from the linear mixed model.

**Model**		**Predicted Weight Change (%, 95%CI)**
**Unadjusted**		−2.15 (−2.30, −2.00)
**Adjusted**		
Male	Under 30	−3.15 (−3.85, −2.45)
Female	Under 30	−1.99 (−2.63, −1.35)
Male	30s	−2.80 (−3.25, −2.36)
Female	30s	−1.65 (−1.99, −1.30)
Male	40s	−2.15 (−2.57, −1.74)
Female	40s	−0.99 (−1.25, −0.74)
Male	50s	−3.07 (−3.47, −2.67)
Female	50s	−1.91 (−2.15, −1.67)
Male	60s	−4.53 (−4.95, −4.11)
Female	60s	−3.37 (−3.65, −3.09)
Male	70+	−4.10 (−4.81, −3.39)
Female	70+	−2.94 (−3.59, −2.29)

**Table 4:** Subscriber survey results showing the self-reported changes and feelings about progress in the program (n = 205).

Question	Response options	n(%)
**What are the biggest changes you have made since starting on Interval Weight Loss? (multiple responses allowed)**	Eating smaller dinner	144 (70%)
	Adopting a long-term weight loss strategy	140 (68%)
	Eating bigger breakfast	139 (68%)
	Eating more frequently	122 (60%)
	Change in food choices	111 (54%)
	Less comfort eating	108 (53%)
	Eating more vegetables	94 (46%)
	Eating less junk food	93 (45%)
	Exercising more	90 (44%)
	Getting more organized	64 (31%)
	Reduced technology use	54 (26%)
	Eating less takeaway	54 (26%)
	Drinking less alcohol	49 (24%)
	Sleeping more	33 (16%)
	Cooking more	33 (16%)
	Other	12 (6%)
**What do you need to work on the most? (multiple responses allowed)**	Exercising more	107 (52%)
	Eating smaller dinner	91 (45%)
	Reducing technology use	78 (38%)
	Changing my long-term weight loss strategy	60 (29%)
	Sleeping more	54 (26%)
	Less comfort eating	50 (25%)
	Getting more organized	44 (22%)
	Eating more frequently	42 (21%)
	Eating more vegetables	40 (20%)
	Drinking less alcohol	36 (18%)
	Change in food choices	32 (16%)
	Eating bigger breakfast	28 (14%)
	Eating less junk food	25 (12%)
	Cooking more	17 (8%)
	Eating less takeaway	9 (4%)
**Which change has been easiest to make?**	Increasing consumption of nature’s treats	56 (28%)
	Change in meal size throughout the day	54 (27%)
	Reducing processed food consumption	47 (23%)
	Exercising	15 (7%)
	Being more organized	10 (5%)
	Change in evening time routine	9 (4%)
	Other	10 (5%)
**Which change has been hardest to make?**	Change in meal size throughout the day	67 (33%)
	Change in evening time routine	58 (28%)
	Exercising	43 (21%)
	Reducing processed food consumption	43 (21%)
	Being more organized	9 (4%)
	Increasing consumption of nature’s treats	8 (4%)
	Other	10 (5%)
**How do you feel about your progress?** (1 = I’m not progressing to 5 = I’m progressing very well)	1	18 (9%)
	2	30 (15%)
	3	90 (44%)
	4	44 (21%)
	5	23 (11%)

**Table 5:** Percentage responses to positive and negative moods prior to and during the IWL program (n = 205).

Mood	Response	Before IWL	During IWL	*p* Value
Balanced				
	Yes	14 (7%)	58 (28%)	<0.001
	No	189 (93%)	147 (72%)	
Calm				
	Yes	10 (5%)	42 (20%)	<0.001
	No	193 (95%)	163 (80%)	
Mostly Happy				
	Yes	89 (44%)	137 (67%)	<0.001
	No	114 (56%)	68 (33%)	
Optimistic				
	Yes	29 (14%)	90 (44%)	<0.001
	No	174 (86%)	115 (56%)	
Anxious				
	Yes	72 (35%)	30 (15%)	<0.001
	No	131 (65%)	175 (85%)	
Busy				
	Yes	103 (51%)	68 (33%)	<0.001
	No	100 (49%	137 (67%)	
Depressed				
	Yes	28 (14%)	8 (4%)	<0.001
	No	175 (86%)	197 (96%)	
Mostly Unhappy				
	Yes	43 (17%)	15 (7%)	<0.001
	No	169 (83%)	190 (93%)	
Stressed				
	Yes	107 (53%)	37 (18%)	<0.001
	No	95 (47%)	168 (82%)	

## Data Availability

Deidentified data may be available for research purposes upon reasonable request and upon obtaining the relevant ethics approval by contacting the Interval Weight Loss program at iwl@intervalweightloss.com.au. The R code for the analysis is available on request from the authors.
